# Glial Hsp70 Protects K^+^ Homeostasis in the *Drosophila* Brain during Repetitive Anoxic Depolarization

**DOI:** 10.1371/journal.pone.0028994

**Published:** 2011-12-12

**Authors:** Gary A. B. Armstrong, Chengfeng Xiao, Jennifer L. Krill, Laurent Seroude, Ken Dawson-Scully, R. Meldrum Robertson

**Affiliations:** 1 Department of Biology, Queen’s University, Kingston, Ontario, Canada; 2 Department of Biological Sciences, Florida Atlantic University, Boca Raton, Florida, United States of America; University of Houston, United States of America

## Abstract

Neural tissue is particularly vulnerable to metabolic stress and loss of ion homeostasis. Repetitive stress generally leads to more permanent dysfunction but the mechanisms underlying this progression are poorly understood. We investigated the effects of energetic compromise in *Drosophila* by targeting the Na^+^/K^+^-ATPase. Acute ouabain treatment of intact flies resulted in subsequent repetitive comas that led to death and were associated with transient loss of K^+^ homeostasis in the brain. Heat shock pre-conditioned flies were resistant to ouabain treatment. To control the timing of repeated loss of ion homeostasis we subjected flies to repetitive anoxia while recording extracellular [K^+^] in the brain. We show that targeted expression of the chaperone protein Hsp70 in glial cells delays a permanent loss of ion homeostasis associated with repetitive anoxic stress and suggest that this is a useful model for investigating molecular mechanisms of neuroprotection.

## Introduction

Neural function is critically dependent on maintaining cellular ion homeostasis which in turn is dependent on an adequate energy supply. Loss of ion homeostasis with consequent depolarization of neurons and glia occurs in response to anoxia (anoxic depolarization; AD) and in healthy tissue complete recovery is possible on return to normoxia within species-specific time limits. Transient loss of ion homeostasis can occur spontaneously and spread through healthy tissue resulting in depolarization (spreading depolarization) and cessation of electrical activity (spreading depression; SD). Whereas *Drosophila* is established as an excellent genetic model for investigating effects of anoxia in whole organisms (e.g.[1], [2], [3], [4]) we know little about how metabolic stress affects ion homeostasis in the fly brain or about mechanisms that could protect brain function in this system.

SD in the mammalian cerebral cortex occurs as a substantial redistribution of ions between intracellular and extracellular compartments coinciding with a near complete depolarization of a sizable proportion of brain cells. The disturbance propagates through grey matter [5] and is associated with many profound disorders of the brain, including migraine with aura [6], traumatic brain injury [7] and stroke [8]; for review see [9]. Although generally considered benign in healthy tissue (but see [10]), when SD is triggered by severe stress, such as focal ischemia or middle cerebral artery occlusion, compromised energy production leads to AD – characterized by cell swelling, dendritic beading and loss of dendritic spines [11], [12], [13], elevated extracellular potassium ion concentration ([K^+^]_o_) [14] and eventually necrosis. Within hours following a stroke, and lasting for days, peri-infarct depolarizations (PIDs) resembling repetitive SD events extend the region of AD around the infarct [15], [16]. A critical difference between PIDs and SD is that PIDs are spontaneous whereas SD occurs in healthy tissue and requires initiating stimuli [8]. Following traumatic brain injury PIDs originate within the injured cortex and propagate outwards, turning into repetitive SD events in the surrounding penumbra [7] and increasing the volume of dead tissue [15], [17]. This increase occurs when energetic costs associated with repetitive SD [18] outstrip the limited energy resources of the metabolically compromised penumbra [19]. It is within the period following a stroke that therapeutic treatments to increase the tissue’s resistance to PID or attenuate their incidence could have significant health benefits. However, cellular mechanisms that modulate repetitive AD or PID and their effect on ion homeostasis are poorly understood.

Fundamental structural and neurophysiological processes are evolutionarily conserved between insects and mammals. Indeed, the hallmarks of SD, including a surge in extracellular potassium ([K^+^]_o_), occur in the thoracic ganglia of the migratory locust (*Locusta migratoria)* in response to anoxia and to metabolic stress induced by compromising the Na^+^/K^+^-ATPase using ouabain [20], [21], [22] suggesting that insects could provide model systems for understanding cortical SD [23]. *Drosophila* recovers well from repetitive anoxia [24] but can be damaged by it when there is insufficient time for full recovery between bouts of anoxia [3]. Thus the powerful molecular genetic tools available for manipulating the nervous system of *Drosophila* make it an ideal model system to explore repetitive anoxic stress in the brain.

## Results

**Figure 1:**
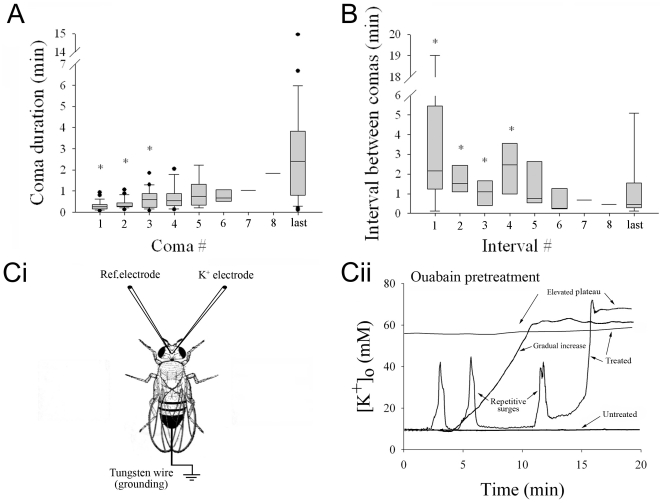
Exposure to ouabain induces repetitive comas coinciding with SD in the fly brain. Coma onset and recovery times were recorded for individual flies which exhibited repeated comas characterized by alternation between activity and immobility (lying on their side). (**A**), Box plots with median, 25^th^ and 75^th^ percentiles (whiskers indicate 10^th^ and 90^th^ percentiles) of the durations of sequential comas (n = 23). (**B**), Box plots (as in A) of the intervals between sequential comas. Asterisks represent significant differences from the duration of the last coma or from the last interval (n = 23; *P*<0.05). Note that the last coma was penultimate to death. (**Ci**), Preparation for monitoring [K^+^]_o_ in the fly brain. The fly was secured in a small bed of wax. A potassium ion-sensitive electrode (K^+^ electrode) was inserted into the neuropil of the brain and referenced to a nearby glass microelectrode recording voltage (Ref. electrode). The preparation was grounded with a tungsten wire in the abdomen. (**Cii**)**,** Representative [K^+^]_o_ recordings from the brain of three ouabain-exposed flies, one fly displaying an elevated plateau, another showing a gradual increase in [K^+^]_o_ and another showing spontaneous surges of [K^+^]_o_ and a loss of K^+^ homeostasis to an elevated plateau. Untreated flies displayed a stable low [K^+^]_o_.

In mammals [25] and locusts [21], [22], [26], [27] mimicking the effect of energetic compromise with the Na^+^/K^+^-ATPase inhibitor ouabain generates spontaneous SD similar to PID. To determine if a similar phenomenon could occur in the fly brain we exposed flies to volatilized ouabain (10 µl of 100 mM; see e.g.[28]) for 1 hour and then videotaped individual fly behaviour. A proportion of flies were noticeably affected by ouabain treatment (65%). Affected flies alternated between activity (walking/flying/grooming) and coma (motionless and unresponsive to touch) which began 78.2±13.2 min following ouabain treatment (n = 23), (Video S1). Successive comas increased in duration and the duration of the last coma was significantly longer than the first three comas (Fig. 1A). Before failure, flies exhibited a median of 5 comas and the last coma was penultimate to death which was determined by examination 24 hrs after the treatment. Coinciding with the increase in coma duration was a decrease in the duration of the interval between comas. The last coma interval was significantly shorter than the first four intervals (Fig. 1B). HS-treated flies (n = 25) were resistant to ouabain treatment with no flies exhibiting comas during 6 hrs of observation. However, 24 hrs after treatment 4 of the 25 HS-preconditioned flies that had been exposed to ouabain were dead. Electrophysiological measurements of [K^+^]_o_ in the brains of flies (Fig. 1Ci) exhibiting repetitive ouabain-induced comas showed recurrent surges of [K^+^]_o_ from a baseline of 13.1±2.8 mM. The small size of the brain precluded measurement of propagation however these events were similar to spontaneous SD-like events evoked by ouabain in locust ganglia (Rodgers et al. 2007). The preparation eventually lost the ability to maintain ion homeostasis and baseline [K^+^]_o_ increased, terminating at a plateau of 54.1±13.9 mM (Paired t-test, *P* = 0.026; n = 12). There was no recovery within the duration of the experiment (Fig. 1Cii). Of 44 similar preparations only 27% showed [K^+^]_o_ surges, whereas 32% showed a gradually rising [K^+^]_o_, 18% showed an elevated plateau [K^+^]_o_ and 23% showed [K^+^]_o_ that remained at low concentrations. These data suggest that spontaneous disturbances in K^+^ homeostasis occur in the fly brain during mimicked energy compromise resulting from inhibition of Na^+^/K^+^ATPase activity. To test if exposure to anoxia resulted in a similar phenomenon we repeatedly exposed flies to nitrogen gas while measuring [K^+^]_o_ in the brain.

**Figure 2:**
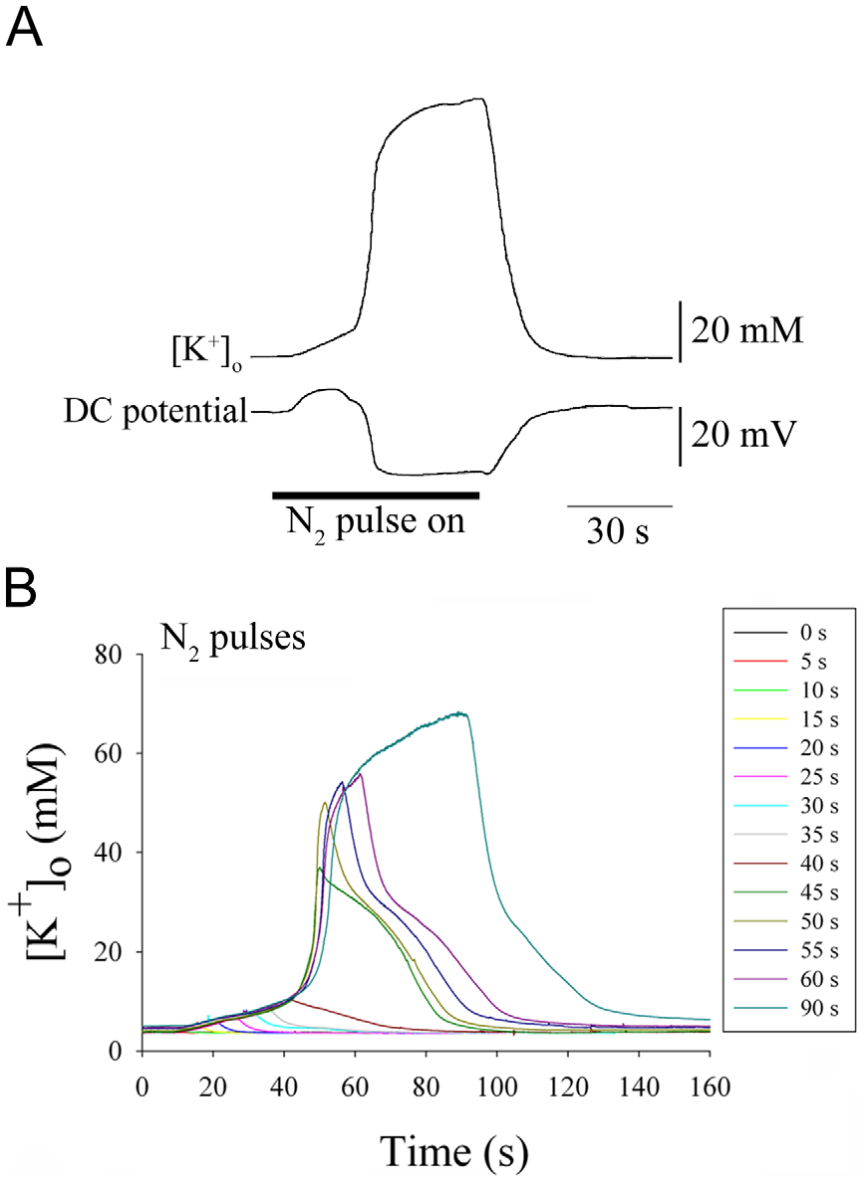
Sample traces of [K^+^]_o_ surges in the fly brain during anoxia induced by exposure to N_2_ gas (**A**)**.** 100% N_2_ gas was delivered through a porous polyethylene substrate. Flow timing and rate was controlled and the gas stream completely covered the animal. A single surge of [K^+^]_o_ in the brain in response to a pulse of N_2_ recorded simultaneously with the extracellular DC potential. Three phases of [K^+^]_o_ increase are evident (slow, fast and slow). (**B**). Surges of [K^+^]_o_ in response to pulses of N_2_ that increased in duration from 5 s to 90 s (traces overlaid and aligned at the start of anoxia). Note that in this preparation durations of N_2_ exposure up to 40 s resulted in small increases of [K^+^]_o_ that recovered quickly. Thereafter there was a surge of rapidly increasing [K^+^]_o_ whose amplitude was dependent on the duration of N_2_ exposure until it reached a plateau that is not illustrated in this figure.

Passing 100 % N_2_ gas over the preparation rapidly and reliably evoked a negative DC potential shift and a simultaneous surge in [K^+^]_o_ in the fly brain which recovered when the fly was returned to normoxia (Fig. 2A). During anoxia the DC potential near the site of the K^+^-sensitive electrode initially increased by 6.5±0.6 mV before a sharp negative deflection of -23.0±1.34 mV (n = 4). These changes in the DC potential resemble those associated with AD [29] and PID [30] in the mammalian brain suggesting increased neuronal firing followed by a rapid onset of cellular depolarization. Increasing the duration of 100% N_2_ exposure from 5 s to 90 s demonstrated a threshold duration for evoking the [K^+^]_o_ surge. Recovery was characterized by two phases of [K^+^]_o_ clearance (n = 5) (Fig. 2B) and suggests the involvement of two clearance mechanisms or two tissue compartments (e.g.[31]). The ability to control the timing of multiple [K^+^]_o_ surges in the brain rapidly and precisely is attractive and it formed the basis for our subsequent experiments.

**Figure 3:**
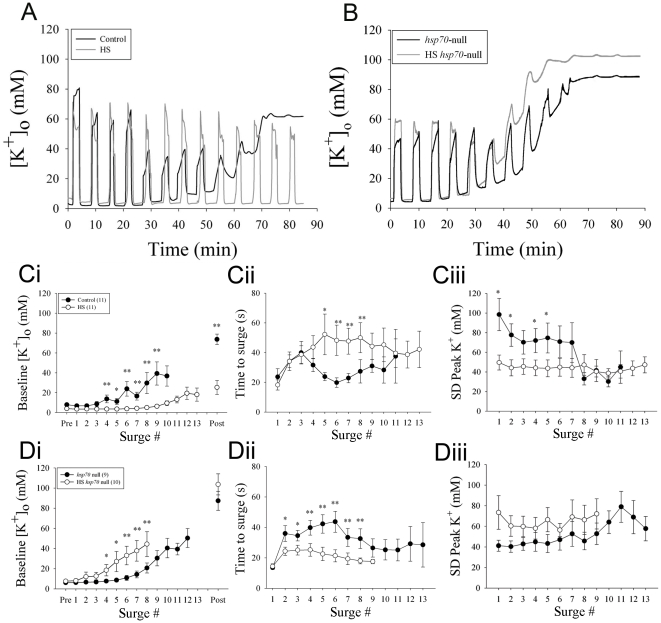
HS preconditioning stabilizes K^+^ homeostasis. Flies were subjected to repetitive anoxia (100% N_2_ gas) in a 2.5 min on/4 min off cycle. In all experiments anoxia rapidly induced a surge in [K^+^]_o_. (**A**), Sample recordings of brain [K^+^]_o_ taken from one control fly (black) and one HS fly (grey). (**B**), Sample recordings of brain [K^+^]_o_ taken from one untreated fly lacking all 6 copies of the *hsp70* gene (*hsp70*-*null*) (black) and one HS-treated *hsp70- null* fly (HS *hsp70*-null) (grey). (**C**), Effects of repetitive anoxia on baseline [K^+^]_o_ (**i**), time to surge (**ii**) and peak [K^+^]_o_ (**iii**) of sequential surges during repetitive exposures to anoxia in control and HS-treated flies. (**D**), Effects of repetitive anoxia on baseline [K^+^]_o_ (**i**), time to surge (**ii**) and peak [K^+^]_o_ (**iii**) of sequential during repetitive exposures to anoxia in *hsp70-null* and HS-treated *hsp70*-null flies. Note that HS-treatment protects the brain from effects of repetitive anoxia but these are exacerbated after HS-treatment in *hsp70-null* flies. Points represent three or more data points and are displayed as means ± standard error. Numbers in parentheses represent sample sizes and single and double asterisks represent significant differences within SD #s (*P*<0.05 and *P*<0.01 respectively).

To assess the deleterious effect of repetitive anoxia we exposed animals to cyclical bouts of 100% N_2_ gas (2.5 min on/4 min off) and measured [K^+^]_o_ in the fly brain for 85 minutes. Under these experimental conditions, repetitive anoxia eventually was associated with a loss of ion homeostasis indicated by a gradual increase of [K^+^]_o_ to a plateau from which we never observed the preparation to recover, though we cannot be certain that longer periods of recovery would be ineffective (Fig. 3A, Ci, Control). In flies preconditioned with HS we observed robust protection of potassium ion homeostasis in the brain. Firstly, these flies displayed a strong ability to recover [K^+^]_o_ to baseline levels between exposures of N_2_ gas whereas untreated flies displayed a poor ability to maintain low [K^+^]_o_ between bouts of anoxia (Fig. 3A,Ci). Secondly, we observed a significant increase in the latency to the [K^+^]_o_ surge during each exposure to anoxia in HS-treated flies (Fig. 3Cii). Thirdly, we observed a reduction in the peak [K^+^]_o_ during surges in HS-treated flies compared to control flies (Fig. 3Ciii). To examine the role of Hsp70 during HS preconditioning we repeated our heat shock protocol in flies lacking all 6 copies of the *hsp70* gene [32] (HS *hsp70-*null). These flies were less able to maintain K^+^ homeostasis compared to untreated *hsp70-*null flies (Fig. 3Di). In addition, HS-treated *hsp70-null* flies were more vulnerable to anoxia and had shorter latencies to the beginning of each surge in [K^+^]_o_ (Fig. 3Dii). Interestingly, under non-HS conditions the *hsp70*-null genotype was associated with an increased stability of [K^+^]_o_ during repetitive anoxia and we suggest that this could be due to mechanisms compensating for the loss of Hsp70 during development, as described for flies deficient in heat shock factor [33]. There was no clear difference of peak [K^+^]_o_ between HS-treated and untreated *hsp70-null* flies (Fig. 3Diii). These data strongly suggest that Hsp70 plays a role in conferring tolerance to repetitive anoxia in the fly brain, specifically by improving the ability of the fly brain to maintain K^+^ homeostasis and delay the onset of K^+^ surges during anoxia. To test this directly we used the GAL4-UAS binary system for tissue-specific expression of Hsp70.

**Figure 4:**
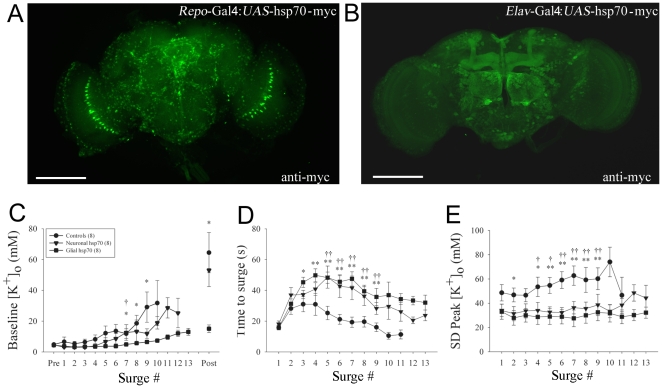
Tissue-specific expression of Hsp70 in glia but not in neurons mitigates the effect of repetitive anoxia on K^+^ homeostasis. *Repo-Gal4* (pan-glial) and *Elav-Gal4* (pan-neuronal) were crossed with *UAS-hsp70-myc* flies. (**A**), Frontal aspect of a fly brain revealing the location of Hsp70-myc in glia when driven by *Repo-Gal4*. Note that glial cells distribute all over the brain tissues including the antennal lobes. The giant glial cells of the optic chiasm are arrayed in a semi-circular fashion in each optic lobe. (**B**), Neuronal Hsp70-myc (green) driven by *Elav-Gal4*. Note prominent staining in the neuronal axons of the mushroom bodies and in the neuropil of antennal lobes. (**C**), Comparison of the effects of repetitive anoxia on baseline [K^+^]_o_ between surges in control flies (including *Repo-Gal4* alone, *Elav-Gal4* alone and *UAS-hsp70* alone) and flies expressing Hsp70 in neurons *(Elav-Gal4:UAS-hsp70*) and in glia (*Repo-Gal4:UAS-hsp70*). (**D**), Effects of repetitive anoxia on time to surge. (e), Effects on peak [K^+^]_o_ reached during exposure to repetitive anoxia.. Points represent three or more data points and are displayed as means ± standard error. Numbers in parentheses represent sample sizes. Asterisks indicate significant differences between control and glial expression; daggers indicate significant difference between control and neuronal expression (2-way RM ANOVA). One asterisk: *P*<0.05. Two asterisks: *P*<0.01. Scale bars in A and B = 100 µm.

A *UAS*-*hsp70* fly line carrying a single copy of the *Drosophila* hsp70Ab gene with a c-myc tag sequence constructed in our laboratory [34] was crossed with GAL4 enhancer trap lines *Repo-* (pan-glial) and *Elav-* (pan-neuronal) *GAL4*. Immunocytochemistry confirmed that Hsp70 was present in the cytoplasm of cells and widely distributed throughout all of the brain tissue of both *Elav-Gal4:UAS-hsp70* and *Repo-Gal4:UAS-hsp70* flies (Fig. 4A,B). We monitored [K^+^]_o_ in *Repo-Gal4:+*, *Elav-Gal4:+* and *UAS-hsp70:+* in addition to *Elav-Gal4:UAS-hsp70* (Neuronal Hsp70) and *Repo-Gal4:UAS-hsp70* (Glial Hsp70) flies during repetitive N_2_ anoxia. The three control groups were found to be not significantly different from each other and for statistical reasons they were combined (Controls). Targeted expression of Hsp70 in glia helped to maintain K^+^ homeostasis between bouts of repetitive anoxia (Fig. 4Ci). The ability to maintain low [K^+^]_o_ in flies expressing Hsp70 in neurons was not significantly different from controls (Fig. 4C). However, the latency to the [K^+^]_o_ surge was significantly longer than controls in flies with Hsp70 expression targeted to glia or neurons (Fig. 4Cii). Lastly we found that flies expressing Hsp70 in either glia or neurons had reduced peak [K^+^]_o_ during repetitive anoxia (Fig. 4Ciii). It is important to note that for measurements of time to surge and peak [K^+^]_o_ (Fig. 4Cii & iii) both neuronal and glial Hsp70 were effective in providing protection. This is in contrast to baseline [K^+^]_o_ which was stabilized by glial Hsp70 more effectively than by neuronal Hsp70.

## Discussion

The utility of *Drosophila* for insight into vertebrate brain function is well-established [35] and the fly has long been used as a model system for the dissection of the genetic basis of tolerance and susceptibility to hypoxia [1], [36]. *Drosophila* are considerably more tolerant to hypoxia than mammals and can survive several hours of exposure to anoxia [2]. Additionally, *Drosophila* has been proposed as a model for studying ischemia and reperfusion injury based on the deleterious effects of repetitive returns to normoxia (every 20 min for 60 s) during maintained anoxia [3]. The linear relationship between the number of reperfusion events and ensuing mitochondrial and neuromuscular failure is reminiscent of the linear relationship between number of PIDs and infarct volume in rats [37]. Mechanisms providing protection of *Drosophila* against hypoxia include upregulation of chaperone proteins. For example Hsp70 and Hsp23 confer protection against constant hypoxia (1.5% O_2_) and targeted expression (heart and regions of the brain) increases survival time [38]. However these Hsps had no protective effects against intermittent hypoxia (a 20 min cycle ramping between 4 min at 1% O_2_ and 4 min of 21% O_2_). Additionally, these previous studies monitored survival of the whole organism and there is almost no information on disturbances in the fly CNS during anoxic coma.

In this study we demonstrate that during anoxic coma the fly brain exhibits a brain disturbance that shares the essential characteristics of AD. Notably, a rapid rise in [K^+^]_o_ during exposure to anoxia which returns to near baseline levels following return to normoxia. We also demonstrate that exposure to the Na^+^/K^+^-ATPase inhibitor ouabain generates repetitive and spontaneous comas in intact freely behaving flies. We attributed the occurrence of these comas to concurrent [K^+^]_o_ surges resulting from compromised Na^+^/K^+^-ATPase activity. Previous work by our lab has documented the occurrence of this phenomenon in the migratory locust (*L. migratoria*) CNS during exposure to ouabain [21], [22], [39] and we have proposed a model for its occurrence [22], [23]. We believe that the spontaneous and repetitive surges in [K^+^]_o_ occurring in the fly brain are the essential distinguishing features of PIDs and sought to investigate exclusively the deleterious effect of repetitive anoxia in the fly brain. To do this we exposed flies to repetitive anoxia (cyclical bouts of 100% N_2_ gas; 2.5 min on/4 min off) and measured [K^+^]_o_ in the fly brain for 85 minutes. This duty cycle was chosen because it was similar to that of the spontaneous [K^+^]_o_ surges we observed in flies pre-treated with ouabain. It was also within the range of frequencies for repetitive PID evident in the rat brain after middle cerebral artery occlusion and reperfusion [40] and for recurrent SD induced by a single stimulus in the brain of Familial Hemiplegic Migraine Type 1 mutant mice [41]. Successive anoxia-induced surges culminated in an inability to restore [K^+^]_o_ to baseline concentrations resulting in an elevated [K^+^]_o_ plateau from which we never observed the preparation to recover (Fig. 3A, Ci), but we cannot be certain that longer periods of recovery would be ineffective at restoring baseline [K^+^]_o_. We hypothesise that the gradual increase in brain [K^+^]_o_ between bouts of anoxia reflects an increasing impairment in the ability of cells to clear [K^+^]_o_. Although anoxia-induced surges were observed in all HS-treated flies, protection against the elevated [K^+^]_o_ plateau was evident. HS-treated flies were better at maintaining low [K^+^]_o_ between bouts of anoxia. This might be due to an improvement in the ability to re-sequester [K^+^]_o_ on return to normoxia and may also account for the delay in producing a surge in [K^+^]_o_ (Fig 3Cii). Previous work has documented that HS preconditioning speeds the rate of recovery of the [K^+^]_o_ surge in locusts [21] and up-regulation of Hsp70, using transgenic mice [42], [43] and virally-mediated gene transfections [44], [45], reduces neural damage in experimental models of stroke. Moreover there is considerable interest in Hsp70 as a therapeutic target in numerous brain pathologies [46], [47]. Conversely, flies lacking Hsp70 displayed a poor ability to maintain K^+^ homeostasis which was worsened by HS preconditioning. Additionally, HS-treated *hsp-70* null flies displayed a poor ability to resist the surge in [K^+^]_o_ (Fig 3 Dii). It should also be noted that *hsp-70* null flies, without HS, displayed longer time to surge values when compared to HS-treated *hsp-70* null flies, however time to surge in *hsp-70* null flies was not significantly different from HS-treated W^1118^ flies. This might be due to mechanisms compensating for the loss of Hsp70 during development, as described for flies deficient in heat shock factor which show an attenuated production of Hsp70 following HS but still acquire synaptic thermotolerance [33].

In a final set of experiments we targeted over expression of Hsp70 in either neurons or glia and exposed flies to repetitive bouts of anoxia. Interestingly, flies over expressing Hsp70 in glia were better at maintaining low [K^+^]_o_ between bouts of anoxia. However flies over expressing Hsp70 in glia or in neurons displayed a significant delay in the time to surge of [K^+^]_o_. These data suggest a testable hypothesis that glial mechanisms have a predominant role in long-term maintenance of ionic gradients under cellular stress (i.e. the vulnerability to repetitive anoxia) whereas both neuronal and glial mechanisms are involved in the acute response of neural tissue to anoxia (i.e the propensity to generate AD).

Much of the long-term disability associated with stroke in mammals is thought to result from an increase in infarct volume during the period following a vascular accident. This increase is associated with PIDs that occur spontaneously and repetitively in the penumbra of vulnerable grey matter around the infarct. In spite of the critical role of events in the peri-infarct zone there is little understanding of how PIDs contribute to damage and consequently no treatments target PIDs. We suggest that repetitive anoxia in the *Drosophila* brain can serve as a model for PIDs and here we show that targeted expression of the chaperone protein Hsp70 in glial cells delays the loss of ion homeostasis associated with repetitive AD. We propose that this model of PID in *Drosophila* affords the opportunity to investigate many of the mechanisms of stroke injury using the powerful and rapid molecular genetic techniques available for the fly.

## Materials and Methods

### Flies lines

All experiments were performed at the Department of Biology at Queen’s University or at the Department of Biological Sciences at Florida Atlantic University. Flies were raised on standard medium (0.01 % molasses, 8.2 % cornmeal, 3.4 % killed yeast, 0.94 % agar, 0.18 % benzoic acid, 0.66 % propionic acid) at 25°C in 60-70 % humidity and reared in a 12 h/12 h (light/dark) cycle with lights on at 0800 h. Flies were maintained at equal densities (approximately 20 flies) in 30 ml plastic vials containing 5 ml of medium.

All electrophysiological experiments were performed on male flies aged 3-7 days post ecdysis or older male flies (14-21 days). The w[1118]; P{w[+m*] = GAL4}repo/TM3,Sb[1] and the w[*]; P{w[+mC] = GAL4-elav.L}3 GAL4 enhancer-trap strains were obtained from the Bloomington *Drosophila* stock center and the UAS Hsp70 lines were constructed in our laboratory [34]. The *hsp70-null* flies were obtained from Kent Golic [32]. Tissue-specific expression of Hsp70 was achieved using the Gal4/UAS system to drive Hsp70 expression in neurons (*Elav-GAL4*) or glia (*Repo-GAL4*). Flies overexpressing Hsp70 were obtained by crossing females carrying the *UAS-hsp70-mcy* construct with males carrying a *GAL4* transgene. To exclude effects due to heterosis, three controls were used: a *UAS-hsp70*/alone control, an *Elav*-*GAL4*/alone, and a *Repo*­-*GAL4*/alone control obtained by crossing, respectively, the UAS strain and the GAL4 strains with w^1118^.

### Heat Shock

Vials containing adult flies (3-7 days post ecdysis) and medium were placed in a humid (∼100 %) incubator at 36°C for 1 h and subsequently removed to allow recovery at room temperature for 1 h.

### Electrophysiology

To facilitate handling, flies were placed in a refrigerator (4°C) for 3 minutes before partial dissection using hemolymph-like solution HL3 [48] and secured on a 0.5 cm diameter bed of wax on either a cover slip or on top of recessed porous platform (6×12 cm, porous polyethylene) capable of delivering a laminar flow of N_2_ gas over the entire fly. The brain was exposed by removing a small piece of cuticle along the dorsal midline of the fly head. K^+^-sensitive microelectrodes were inserted through the sheath into the brain to measure [K^+^]_o_.

K^+^-sensitive microelectrodes were fashioned from non-filamented glass pipettes (1 mm diameter; World Precision Instruments Inc., Sarasota, FL, USA) that were cleaned with methanol (99.9 %) and dried on a hot plate, then pulled to form a low resistance (6–8 MΩ) tip. The microelectrodes were silanized by exposure to dichlorodimethylsilane (99 %) (Sigma-Aldrich) vapour while baking on a hot plate (100°C) for 1 h. After cooling, the microelectrodes were first back-filled to the tip with Potassium Ionophore I-Cocktail B (5 % Valinomycin; Sigma-Aldrich) to form an artificial membrane permeable to K^+^ and then back-filled with 500 mM KCl. The tips of the K^+^-sensitive microelectrodes were suspended in distilled water until experimentation. Reference electrodes were made by pulling a filamented pipette (1 mm diameter; World Precision Instruments Inc., Sarasota, FL, USA) to form a low resistance (6–8 MΩ) tip and were back-filled with 3 M KCl.

The K^+^-sensitive and reference microelectrodes were inserted into an electrode holder with a chloride-coated silver wire and connected to a DUO 773 two-channel intracellular/extracellular amplifier (World Precision Instruments) and calibrated at room temperature (20°C). Two KCl solutions (15 and 150 mM) were used to determine the voltage change needed to establish [K^+^]_o_ (mM) using the Nernst equation. Electrode sensitivities ranged from 50 to 60 mV for a 10-fold change in K^+^ concentrations (e.g. 15 and 150 mM) and electrodes which gave voltage changes outside of this range were discarded. The voltage of the K^+^ -sensitive electrode is logarithmically related to the potassium concentration [21].

### Intact animal ouabain treatment

W^1118^ males were aged to 14-21 days. Flies were placed in an air-tight sealed vial with 10 µl of 100 mM Ouabain (Sigma-Aldrich) in DMSO (BDH) for one hour in the dark at room temperature prior to video analysis [49]. Comas were identified as periods of time longer than 5 s when individuals were lying on their sides, motionless and unresponsive to touch or vibration. Flies were monitored for 6 hrs and coma start and stop times were recorded for each individual fly, up to and including the time to permanent coma onset.

### Anoxia test

AD was induced rapidly and reliably by passing a stream of N_2_ over the preparation. The majority of experiments used a protocol of repeating 2.5 minutes anoxia followed by 4 minutes recovery to measure the consequences of repetitive AD. To test the effects of increasing the duration of anoxia exposure on the shape of [K^+^]_o_ surges we slowly extended the duration from 5 to 90 s in 5 s steps.

### Immunostaining

Immunohistochemistry was conducted according to a previously described protocol [50]. Briefly, dissected adult brains of 7-day old male flies were fixed in freshly prepared 4 % formaldehyde for 20 min at room temperature. The tissues were then washed and saturated in 5 % goat serum (Sigma) for 1 h at room temperature. Incubation with primary antibodies was performed at 4°C for 48 h with a rabbit anti-c-myc (GenScript) at 1∶50. Following the wash, tissues were incubated in secondary antibodies at 4°C for 48 h. Secondary antibodies were DyLight 488 conjugated goat anti-rabbit IgG (Jackson ImmunoResearch) at 1∶500. The incubation tubes were wrapped with aluminum foil to keep the tissue in the dark. After three separate washes, tissues were re-suspended in 200 µl SlowFade Gold Antifade reagent (Invitrogen) and mounted on slides. Confocal images were taken with a Carl Zeiss LSM 710 NLO Laser Scanning Confocal/Multiphoton Microscope and processed with LSM software Zen 2009 (Carl Zeiss).

### Statistical analysis

SigmaPlot 11.0 integrated with SigmaStat 3.1 was used to assess data groupings for significance. Statistical analyses used one-way and two-way repeated measures ANOVA, followed by a post-hoc Tukey multiple comparison test. For non-parametric tests a Kruskal-Wallis one way ANOVA on ranks was performed. For before and after experiments paired t-tests were performed. Significance was assessed at *P*<0.05 (single asterisks or daggers) however the majority of *P*-values are less than 0.01 (double asterisks or daggers).

## Supporting Information

Video S1 Footage of a adult fly exhibiting repetitive comas. The fly was exposed to volatilized ouabain (10 µl of 100 mM) for 1 hr prior to videotaping.
(WMV)
